# A little education goes a long way: Decreasing antibiotics for community-acquired pneumonia in COVID-19 patients

**DOI:** 10.1017/ash.2022.62

**Published:** 2022-05-16

**Authors:** Ravi Tripathi, Rohini Dave, Elizabeth Eden, Jacqueline Bork

## Abstract

**Background:** Antibiotic use was common in patients with suspected or confirmed COVID-19 infection; however, data emerged demonstrating low rates of bacterial coinfection (6%–10%). Antimicrobial stewardship best practice was challenged during this time, requiring new strategies and education to limit the inappropriate use of antibiotics. At the Veterans’ Affairs Maryland Healthcare System, we evaluated the use of community acquired pneumonia (CAP) specific antibiotics in COVID-19–positive patients after successive interventions. **Methods:** We conducted a pre–post evaluation of common CAP antibiotics (ceftriaxone IV/IM, cefpodoxime PO, azithromycin PO/IV, ampicillin/sulbactam IV, amoxicillin-clavulanate PO, levofloxacin) during the COVID-19 pandemic. The preintervention period was April–October 2020 and the postintervention period was November 2020–April 2021. During the preintervention period, intervention A was carried out as follows: (1) inpatient weekly virtual interdisciplinary COVID-19 rounds were led by an antimicrobial stewardship champion, (2) *χ*procalcitonin was implemented in clinical decision making, and (3) inpatient audit and feedback of active antibiotics was conducted by the antimicrobial stewardship team. In the postintervention period, intervention B was added as follows: (1) weekly educational COVID-19 virtual seminars were conducted for providers, and (2) targeted education was provided to emergency department and hospitalist directors. Comparisons of the proportions of antibiotics prescribed were made between the pre- and postintervention periods using X^2^ statistic, and data were stratified by location. The rates of CAP antibiotic prescription per 100 COVID-19–positive patients were also compared using Poisson distribution. **Results:** During the study period, 814 unique patients had COVID-19 infection: 182 (22.4%) patients admitted to the acute-care center, 66 (8.1%) long-term care residents, and 566 (69.5%) were managed outside the hospital. Of these 814 patients, 211 (25%) were prescribed a CAP antibiotic. Of the antibiotics prescribed, 223 (61%) were ceftriaxone, cefpodoxime, amoxicillin-clavulanate, or ampicillin-sulbactam; 123 (34%) were azithromycin; and 16 (4.4%) were levofloxacin. We observed a decrease in the frequency of all antibiotic prescriptions after intervention B was added: 32% (86 of 273) vs 23% (125 of 541) (*P* = .01). Decreases in antibiotic prescriptions were observed in all locations: acute care (57% vs 44%), long-term care (53% vs 41%) and outpatient care (19% vs 15%). The rates of CAP antibiotic prescribing per 100 COVID-19–positive patients were 114 in the preintervention period and 45 in the postintervention period, a rate difference of −70 antibiotics per 100 COVID-19–positive patients (*p* **Conclusions:** Curbing antibiotic use for CAP indication during the COVID-19 pandemic was a challenge. A multifaceted approach focusing on education was an impactful intervention leading to significant decreases in antibiotic prescribing despite COVID-19 cases increasing.

**Funding:** None

**Disclosures:** None

